# Analysis of stromal PDGFR-β and α-SMA expression and their clinical relevance in brain metastases of breast cancer patients

**DOI:** 10.1186/s12885-023-10957-5

**Published:** 2023-05-22

**Authors:** Md Rashedunnabi Akanda, Eun-Jung Ahn, Yeong Jin Kim, S M Abdus Salam, Myung-Giun Noh, Tae-Kyu Lee, Sung Sun Kim, Kyung-Hwa Lee, Kyung-Sub Moon

**Affiliations:** 1 Department of Pathology, Chonnam National University Research Institute of Medical Science, Chonnam National University Hwasun Hospital and Medical School, 322 Seoyang-ro, Hwasun-eup, Hwasun-gun, 58128 Jeollanam-do South Korea; 2 Department of Neurosurgery, Chonnam National University Research Institute of Medical Science, Chonnam National University Hwasun Hospital and Medical School, 322 Seoyang-ro, Hwasun-eup, Hwasun-gun, 58128 Jeollanam-do South Korea; 3 Department of Pharmacology and Toxicology, Sylhet Agricultural University, Sylhet, Bangladesh; 4 BioMedical Sciences Graduate Program (BMSGP), Chonnam National University, Hwasun, Jeollanam-do South Korea

**Keywords:** Brain metastasis, Cancer-associated fibroblasts, α-smooth muscle actin, Platelet-derived growth factor receptor-β, Prognosis, Triple negative breast neoplasms

## Abstract

**Background:**

Breast cancer brain metastasis (BCBM) is a growing therapeutic challenge and clinical concern. Stromal cancer-associated fibroblasts (CAFs) are crucial factors in the modulation of tumorigeneses and metastases. Herein, we investigated the relationship between the expression of stromal CAF markers in metastatic sites, platelet-derived growth factor receptor-beta (PDGFR-β), and alpha-smooth muscle actin (α-SMA) and the clinical and prognostic variables in BCBM patients.

**Methods:**

Immunohistochemistry (IHC) of the stromal expression of PDGFR-β and α-SMA was performed on 50 cases of surgically resected BCBM. The expression of the CAF markers was analyzed in the context of clinico-pathological characteristics.

**Results:**

Expression of PDGFR-β and α-SMA was lower in the triple-negative (TN) subtype than in other molecular subtypes (*p* = 0.073 and *p* = 0.016, respectively). And their expressions were related to a specific pattern of CAF distribution (PDGFR-β, *p* = 0.009; α-SMA, *p* = 0.043) and BM solidity (*p* = 0.009 and *p* = 0.002, respectively). High PDGFR-β expression was significantly related to longer recurrence-free survival (RFS) (*p* = 0.011). TN molecular subtype and PDGFR-β expression were independent prognostic factors of recurrence-free survival (*p* = 0.029 and *p* = 0.030, respectively) and TN molecular subtype was an independent prognostic factor of overall survival (*p* < 0.001).

**Conclusions:**

Expression of PDGFR-β in the stroma of BM was associated with RFS in BCBM patients, and the clinical implication was uniquely linked to the low expression of PDGFR-β and α-SMA in the aggressive form of the TN subtype.

**Supplementary Information:**

The online version contains supplementary material available at 10.1186/s12885-023-10957-5.

## Background

Breast cancer (BC) is one of the most common malignancies in women and the second leading cause of metastases in the brain [1]. BC brain metastases (BCBM) have a poor prognosis and restricted therapeutic response due to the minimal blood-brain barrier (BBB) penetration of chemotherapeutic and targeted agents [2, 3]. Existing conventional therapies including surgical resection, whole-brain radiation therapy (WBRT), stereotactic radiosurgery (SRS), targeted drugs, and immune checkpoint inhibitors are seldom curative in BM [4]. Consequently, the median survival of BM patients is about a year after diagnosis [5].

The characteristic of cancer metastases and progression is determined by the reciprocal and dynamic interactions between the cancer cells and stromal cells as part of the tumor microenvironment (TME) in the host tissue; this interaction supports in cancer cell survival, invasion, and distant metastasis [6, 7]. TME, particularly the stromal components, is a vital and critical part of the contemporary development of BC therapeutic strategies [8]. Stromal cancer-associated fibroblasts (CAFs), are considered the most vital TME elements and are emerging therapeutic targets for BCBM. Although CAF populations are presumed to be heterogeneous in TME and have been known to promote tumorigenesis and metastasis, their precise functions in systemic or metastatic cancers are not fully elucidated [9].

Previous studies have shown that various CAFs subtypes are pro- or anti-tumorigenic, with their biological actions reliant on their specific tumor conditions [10, 11]. Specific CAF subtypes may affect tumor growth and/or inhibition, possibly according to the primary tumor types and locations [11]. The depletion of α-SMA in the tumor stroma prompts immunosuppression and cancer progression with shortened survival in pancreatic cancer patients [12]. A high expression of α-SMA is associated with longer overall survival (OS) after tumor resection in hepatocellular carcinoma and pancreatic cancer [13]. Besides, the abundant expression of stromal fibroblast activation protein (FAP) is also related to longer OS and disease-free survival (DFS) in patients with invasive ductal carcinoma of the breast [14]. These studies, therefore, argued that stromal CAFs may slow the progression of the tumor and thus increase the patient’s survival.

Numerous CAF markers have been used in clinical and biological studies of CAFs in human cancer so far [15], but the current study was conducted with a focus on PDGFR-β and α-SMA. We investigated the expression level of these markers of the stromal CAFs in BCBMs and evaluated he correlation of their expressions with clinico-pathological variables. In addition, we analyzed the recurrence-free survival (RFS) and overall survival (OS) rates according to the expression levels of CAF markers and clinico-pathological variables.

## Methods

### The study subjects and clinico-radiological data

The study included 50 samples from patients with BCBM who underwent craniotomy and tumor removal at the Chonnam National University Hwasun Hospital between 2004 and 2020. Only 18 out of 50 cases had a matched primary tumor sample. All the specimens were obtained from the archives of Pathology department. Experienced pathologists examined and assessed the selection of the slides and pathological parameters (SSK and KHL).

To define the clinical and radiological variables of the enrolled BCBM patients, we evaluated their clinical data at the time of the BM diagnosis. For this study, we analyzed clinical and radiological variables that have been established to be associated with brain metastasis (BM), based on our previous investigations [16, 17]. Clinical variables include age, presenting symptoms & neurological signs, the time interval from BC diagnosis to BM, the molecular subtype of BC, Karnofsky performance score (KPS), treatment modality after BM resection and date of death. Based on the initial and follow-up magnetic resonance imaging (MRI) and computed tomography (CT) scans, we analyzed the radiological variables, including location, size and tumor nature (solid *vs.* cystic or mixed) of resected BM, the multiplicity of BM, perilesional edema, and resection degree. The presence of extracranial metastasis was also evaluated using positron emission tomography (PET) scan or systemic CT or MRI. RFS in cases with brain tumor was generally calculated from the date of surgery to the date of local recurrence (new lesion on operation site in the cases with gross total resection, or progression in the cases with non-gross total resection). For statistical significance in survival rate, however, RFS was calculated only in 38 cases, excluding 12 cases in which partial to subtotal resection was performed. OS was calculated from the date of surgery to the date of recurrence or death, or the last follow-up visit of a living patient. Written informed consent for the use of clinical information and pathological specimens was obtained from patients or their legal surrogates. The study was approved by the Institutional Review Board of Chonnam National University Hwasun Hospital (CNUHH-2019–218).

### Immunohistochemistry for BM samples

**Fig. 1:**
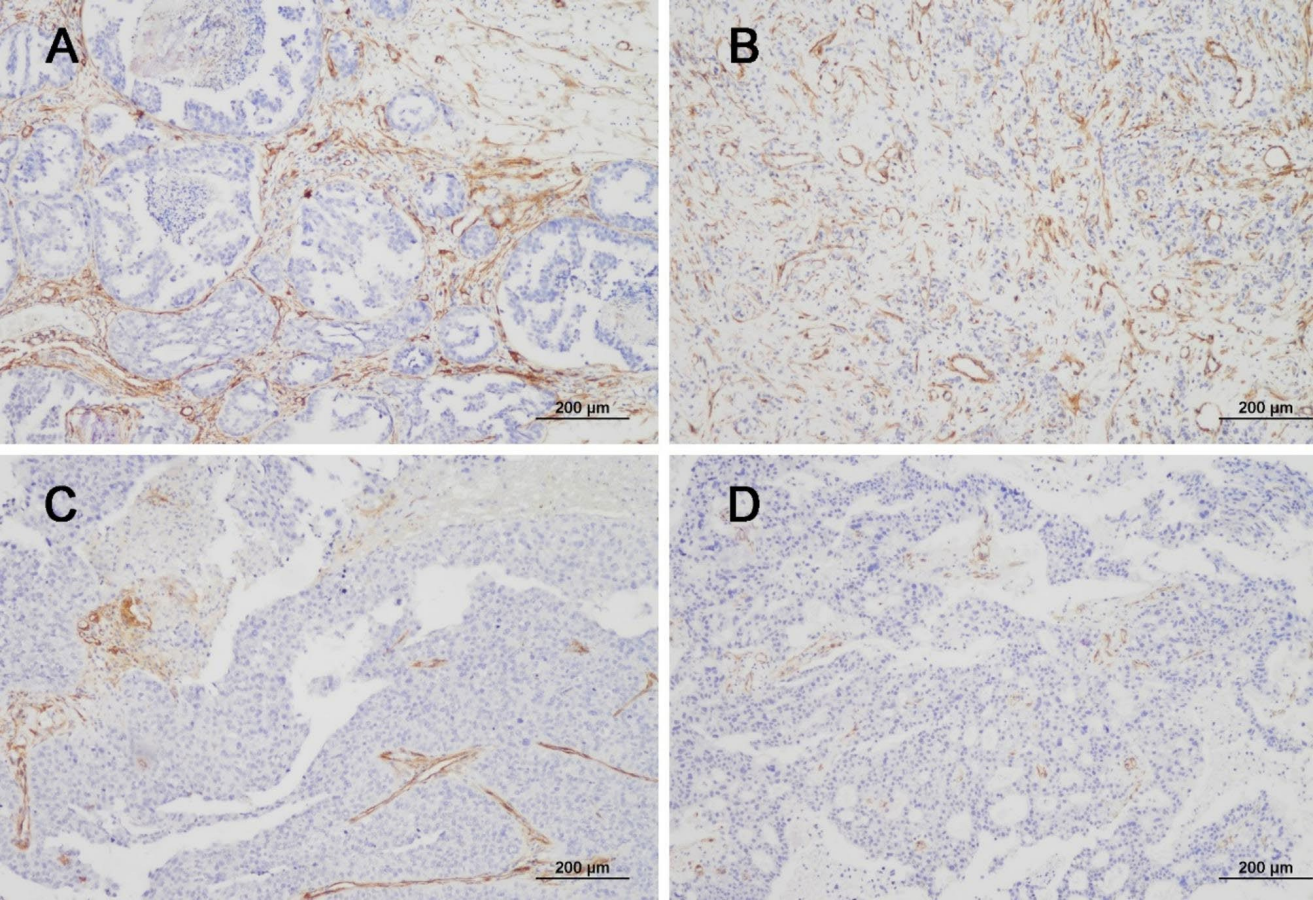
Comparison of CAF marker expression by pattern and score. (**A**) A representative case with a combination of high PDGFR-β expression and CAF pattern A was shown, and its molecular subtype was luminal A-like. (**B**) A representative case with a combination of high PDGFR-β expression and CAF pattern B was shown, and its molecular subtype was HER2-positive. (**C**) A representative case with a combination of low PDGFR-β expression and CAF pattern C was shown, and its molecular subtype was triple-negative. The triple negative type had a high proportion of low expression. (**D**) The case was composed of a combination of low PDGFR-β expression, CAF pattern 4, and luminal A subtype. In luminal-A type, the proportion of low expression was relatively low

Immunohistochemistry (IHC) was performed as previously described [18]. Tissue Sect. (3 μm thick) were subjected to IHC using a Bond-max autostainer (Leica Microsystems, Buffalo Grove, IL, USA). The following antibodies were used: PDGFR-β (1:400 dilution; catalog no. ab32570; Abcam, Cambridge, UK) and α-SMA (1:100 dilution; catalog no. ab5694; Abcam). IHC slides were assessed by pathologists (SSK and KHL), who were blinded to the clinical details. The distribution of CAFs varied significantly according to the molecular subtype and could be grouped into three patterns **(**Fig. 1**).** Briefly, in pattern A, CAFs were located around medium-to-large tumor cell clusters. The CAFs in pattern B encased individual cancer cells or intermingled closely with small tumor nests. In pattern C, the CAFs were scarcely present in the tumor tissue. The scores of immunostaining in stromal CAFs were given according to the relative ratio of the area stained by the CAF markers to the area of the tumor cells **(**Fig. 1**)**: 0, no staining; 1, ≤ 3%; 2, ≤ 10%; and 3 > 10%. Since PDGFR-β and α-SMA are well-known pericyte markers and tend to stain strongly in vascular structures, great care was taken to exclude vessels during the interpretation of the staining area. We assessed the expression levels of PDGFR-β and α-SMA and determined the optimal cut-off value to represent statistical significance by grouping scores of 0, 1, and 2 together and comparing them to a score of 3. Therefore, the cases with scores 0, 1, and 2 were categorized into a low expression group, and the cases with score 3 into a high expression group.

### Statistical analyses

All data were analyzed using SPSS version 23.0 software for Windows (SPSS, Chicago, IL, USA). Comparisons between the expression of CAF markers and clinico-radiological variables were performed using the chi-square test or Fisher exact test as appropriate. The *kappa* value was obtained to show the concordance of expression between different CAF markers or in matched pair samples, and the Wilcoxon signed rank test was used to show the difference in expression level between groups. The effects of clinico-radiological variables and expression and patterns of CAF markers on RFS and OS were determined using the Kaplan–Meier method with log-rank tests. The cox regression model was used for multivariate analysis of patients’ survival. *P* values < 0.05 were considered statistically significant.

## Results

### Correlation between expressions of PDGFR-β/α-SMA in stromal CAFs and clinico-radiological characteristics of BCBM patients

**Fig. 2:**
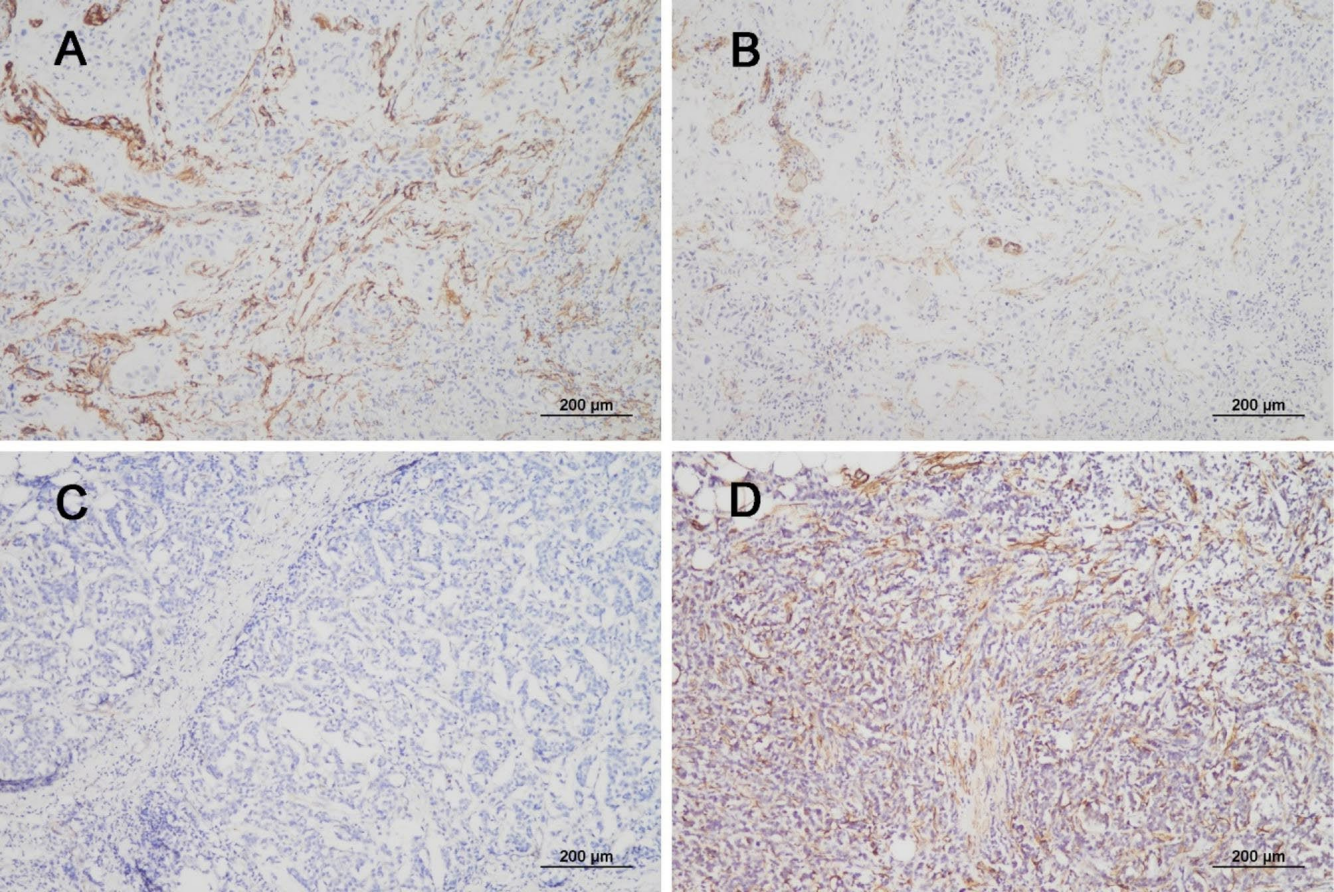
Comparison of PDGFR-β and α-SMA expression in the same case. In the same BM case, the expression of PDGFR-β (**A**) at score 3 and α-SMA (**B**) at score 2 was compared. Comparison of the two markers in 50 BM cases, showed a moderate level of agreement (*kappa* value = 0.684). The expression of PDGFR-β (**C**) at score 1 and α-SMA (**D**) at score 3 in the same primary BC was compared. The expression of the two markers showed no agreement in 18 primary BC samples (*kappa* value = 0.091)

The clinico-radiological and pathological characteristics of the 50 cases of BCBM are summarized in Table 1. In molecular subtypes, 10 cases (20%) were luminal A, 6 cases (12%) luminal B, 19 cases (38%) HER2-positive, and 15 (30%) were TN subtypes. In IHC, PDGFR-β and α-SMA, characteristic biomarkers of CAFs, were specifically stained in the stromal portion, not in the tumor portion. The expression of PDGFR-β was scored as follows; score 1 in 11 cases, score 2 in 16 cases, and score 3 in 23 cases. In α-SMA, 9 cases were scored as 1, 18 cases as 2, and 23 cases as 3. In a comparison of the markers in the same case, the score of expression was consistent in the majority of BM cases and showed a moderate level of agreement (*kappa* value = 0.684) (Fig. 2A-B & Table 2). PDGFR-β and α-SMA expressions were similarly examined in 18 matched primary tumor samples out of 50 cases. Compared to BCBM samples, the expression intensity of PDGFR-β and α-SMA was relatively high in the primary tumor samples without statistical significance (*p* = 0.177 and 0.132, respectively), and the concordance was low (*kappa* value = 0.170 and 0.148, respectively) (Supplementary Tables 1–2). When comparing the expression of PDGFR-β and α-SMA in 18 cases of primary BC samples, the expression of the two markers showed no agreement (*kappa* value = 0.091) (Fig. 2C-D & Supplementary Table 3). It is generally known that a *kappa* value of 0.8 or more indicates a strong level of agreement, 0.6–0.79 indicates a moderate level, and 0.2 or less indicates no agreement [19].

**Table 1:** Clinico-radiological and pathological features of the patients with BCBM

Variable		No.	Percent
Age (year)	< 60	35	70%
	≥ 60	15	30%
Sex	Female	49	98%
	Male	1	2%
Molecular type	Luminal A	10	20%
	Luminal B	6	12%
	HER2	19	38%
	T**N**	15	30%
CAF pattern	Type A	24	48%
	Type B	20	40%
	Type C	6	12%
PDGFR-β	Low	27	54%
	High	23	46%
α-SMA	Low	27	54%
	High	23	46%
Tumor location	Supratentorial	37	74%
	Infratentorial	13	26%
Extracranial Mets	Absent	12	24%
	Present	38	76%
BM symptom	Mild to moderate	31	62%
	Severe	19	38%
KPS	≥ 80	32	64%
	< 80	18	36%
BM multiplicity	Single	24	48%
	Multiple	26	52%
BM development	Metachronous	49	98%
	Synchronous	1	2%
BM size	< 4 cm	22	44%
	≥ 4 cm	28	56%
Peritumoral edema	Mild to moderate	27	54%
	Severe	23	46%
Tumor nature	Solid	27	54%
	Cystic or mixed	23	46%
Resection degree	Gross total	38	76%
	Partial to subtotal	12	24%
Postoperative RT/GKS	Absent	16	32%
	Present	34	68%

BC, breast cancer; BM, brain metastasis; α-SMA, alpha-smooth muscle actin; CAF, cancer-associated fibroblast; GKS, gamma knife radiosurgery; KPS, Karnofsky performance score; Mets, metastasis; PDGFR-β, platelet-derived growth factor receptor-beta; RT, radiation treatment; TN, triple negative

**Table 2:** Comparison of immunohistochemical expressions of PDGFR-β and α-SMA in stromal CAFs.

		α-SMA			Total	*Kappa* value
		Score 1	Score 2	Score 3		
PDGFR-β	Score 1	8	3	0	11	0.684
	Score 2	1	12	3	16	
	Score 3	0	3	20	23	
Total		9	18	23	50	

α-SMA, alpha-smooth muscle actin; CAF, cancer-associated fibroblast; PDGFR-β, platelet-derived growth factor receptor-beta

The correlation between the expression of PDGFR-β and α-SMA and clinico-radiological variables in 50 enrolled patients is summarized in Table 3. Within the various clinico-radiological characteristics, the expression of these markers was correlated with molecular subtypes. In the aggressive TN subtype, the expression of PDGFR-β (*p* = 0.073) and α-SMA (*p* = 0.016) was significantly low compared with other molecular subtypes. High expression of these markers was related to a specific type B pattern of CAF distribution (*p* = 0.005 in PDGFR-β, *p* = 0.028 in α-SMA). Interestingly, the high expression of these markers was associated with the tumor with solid tumors (*p* = 0.009 in PDGFR-β, *p* = 0.001 in α-SMA). Collectively, PDGFR-β and α-SMA expressions were significantly related to the pattern of CAF distribution, molecular subtypes, and tumor nature of BM.

**Table 3:** Correlation between expression of PDGFR-β/α-SMA and clinico-radiological variables in 50 patients with BCBM

Variables		No.	PDGFR-β expression		*p* value	α-SMA expression		*p* value
			Low	High		Low	High	
Age (year)	< 60	35	20 (57%)	15 (43%)	0.496	20 (57%)	15 (43%)	0.496
	≥ 60	15	7 (47%)	8 (53%)		7 (47%)	8 (53%)	
Sex	Female	49	27 (55%)	22 (45%)	0.460	27 (55%)	22 (45%)	0.460
	Male	1	0 (0%)	1 (100%)		0 (0%)	1 (100%)	
Molecular type	Non-TN	35	16 (46%)	19 (54%)	0.073	15 (43%)	20 (57%)	0.016
	T**N**	15	11 (73%)	4 (27%)		12 (80%)	3 (20%)	
CAF pattern	Type B	20	6 (30%)	14 (70%)	0.005	7 (35%)	13 (65%)	0.028
	Other type	30	21 (70%)	9 (30%)		20 (67%)	10 (33%)	
Tumor location	Supratentorial	37	19 (51%)	18 (49%)	0.526	19 (51%)	18 (49%)	0.526
	Infratentorial	13	8 (62%)	5 (38%)		8 (62%)	5 (38%)	
Extracranial Mets	Absent	12	6 (50%)	6 (50%)	0.750	5 (42%)	7 (58%)	0.325
	Present	38	21 (55%)	17 (45%)		22 (58%)	16 (42%)	
BM symptom	Mild to moderate	31	17 (55%)	14 (45%)	0.879	17 (55%)	14 (45%)	0.879
	Severe	19	10 (53%)	9 (47%)		10 (53%)	9 (47%)	
KPS	≥ 80	32	19 (59%)	13 (41%)	0.309	19 (59%)	13 (41%)	0.309
	< 80	18	8 (44%)	10 (56%)		8 (44%)	10 (56%)	
BM multiplicity	Single	24	13 (54%)	11 (46%)	0.982	11 (46%)	13 (54%)	0.266
	Multiple	26	14 (54%)	12 (46%)		16 (62%)	10 (38%)	
BM development	Metachronous	49	27 (55%)	22 (45%)	0.460	27 (55%)	22 (45%)	0.460
	Synchronous	1	0 (0%)	1 (100%)		0 (0%)	1 (100%)	
BM size	< 4 cm	22	12 (55%)	10 (45%)	0.945	11 (50%)	11 (50%)	0.615
	≥ 4 cm	28	15 (54%)	13 (46%)		16 (57%)	12 (43%)	
Peritumoral edema	Mild to moderate	27	16 (59%)	11 (41%)	0.419	18 (67%)	9 (33%)	0.052
	Severe	23	11 (48%)	12 (52%)		9 (39%)	14 (61%)	
Tumor nature	Solid	27	10 (37%)	17 (63%)	0.009	9 (33%)	18 (67%)	0.001
	Cystic or mixed	23	17 (74%)	6 (26%)		18 (78%)	5 (22%)	

BC, breast cancer; BM, brain metastasis; α-SMA, alpha-smooth muscle actin; CAF, cancer-associated fibroblast; GKS, gamma knife radiosurgery; KPS, Karnofsky performance score; Mets, metastasis; PDGFR-β, platelet-derived growth factor receptor-beta; RT, radiation treatment; TN, triple negative

### Prognostic significance of PDGFR-β/α-SMA in stromal CAFs and clinico-radiological characteristics concerning survival outcomes

**Fig. 3:**
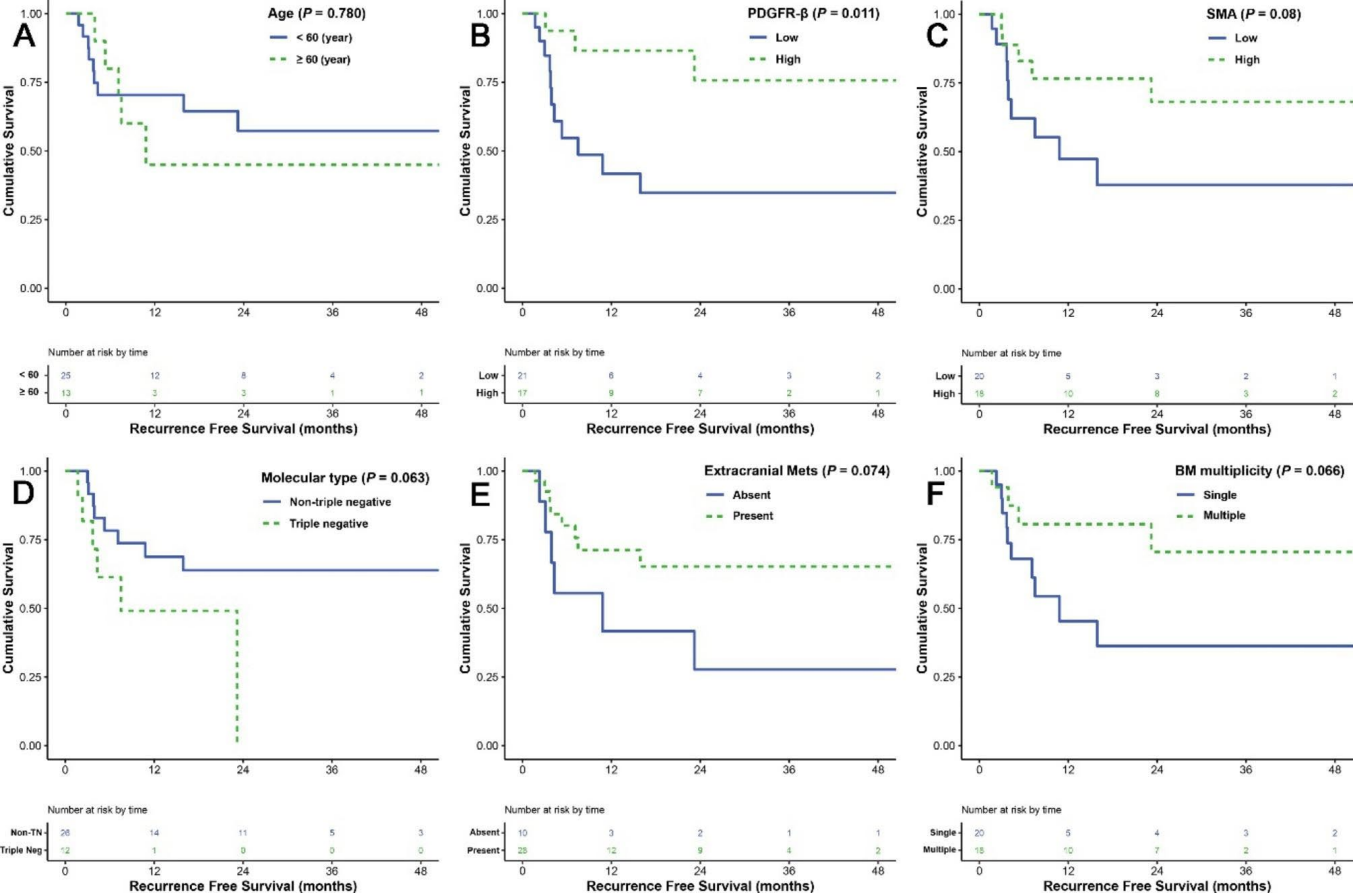
Recurrence-free survival (RFS) analyses using the Kaplan-Meier estimator and log-rank test were performed according to age (**A**), PDGFR-β expression (**B**), α-SMA expression (**C**), molecular subtype of BCBM (**D**), presence of extracranial metastasis (**E**), and multiplicity of BM lesions (**F**)

We analyzed the RFS and OS of the patients based on the CAF-markers expression and clinico-pathological variables. As shown in Fig. 3 & Table 4, RFS analyses using the Kaplan-Meier estimator and log-rank test revealed that higher expression of PDGFR-β was significantly related to longer RFS (*p* = 0.011, 31.2 m in low expression *vs.* 50.1 m in the high expression). A similar trend was found in the analysis of α-SMA expression with marginal significance (*p* = 0.080, 28.3 m in low expression *vs.* 56.4 m in the high expression). As in presumption, the TN subtype was correlated with shorter RFS (13.5 m in TN subtype *vs.* 52.5 m in non-TN subtypes, *p* = 0.063). On multivariate analysis, high expression of PDGFR-β (*p* = 0.011, hazard ratio [HR] 0.228, 95% confidence index [CI] 0.060–0.868) and TN molecular subtype (*p* = 0.029, HR 4.202, 95% CI 1.158–15.256) were independent predictors of RFS.

**Table 4:** Univariate and multivariate analysis for recurrence-free survival (RFS) predictors in 38 patients with BCBM that were removed by gross total resection

Characteristics		No.	Mean (months)	P-value (univariate)	P-value (multivariate)	Hazard ratio	95% CI
Age (year)	< 60	25	48.5	0.780	0.275	1	0.571–7.164
	≥ 60	13	31.8			2.023	
Sex	Female	37	---	---	---		
	Male	1	---				
Molecular type	Non-TN	26	52.5	0.063	0.029	1	1.158–15.256
	T**N**	12	13.5			4.202	
CAF pattern	Type B	15	43.2	0.424	0.254	1	0.112–1.782
	Others	23	41.8			0.447	
PDGFR-β	Low	21	31.2	0.011	0.030	1	0.060–0.868
	High	17	50.1			0.228	
α-SMA	Low	20	28.3	0.080	---		
	High	18	56.4				
Tumor location	Supratentorial	29	45.3	0.968	---		
	Infratentorial	9	25.6				
Extracranial Mets	Absent	10	28.0	0.074	0.072	1	0.106–1.099
	Present	28	44.0			0.341	
BM symptom	Mild to moderate	24	48.3	0.500	---		
	Severe	14	33.4				
KPS	≥ 80	25	44.1	0.806	---		
	< 80	13	38.5				
BM multiplicity	Single	20	32.9	0.066	---		
	Multiple	18	48.1				
BM development	Metachronous	37	---	---	---		
	Synchronous	1	---				
BM size	< 4 cm	19	39.0	0.428	---		
	≥ 4 cm	19	41.3				
Peritumoral edema	Mild to moderate	16	38.9	0.676	---		
	Severe	22	43.7				
Tumor nature	Solid	23	45.1	0.919	---		
	Cystic or mixed	15	38.6				
Postoperative RT/GKS	Absent	9	22.8	0.249	---		
	Present	29	42.2				

BC, breast cancer; BM, brain metastasis; α-SMA, alpha-smooth muscle actin; CAF, cancer-associated fibroblast; GKS, gamma knife radiosurgery; KPS, Karnofsky performance score; Mets, metastasis; PDGFR-β, platelet-derived growth factor receptor-beta; RT, radiation treatment; TN, triple negative

**Fig. 4:**
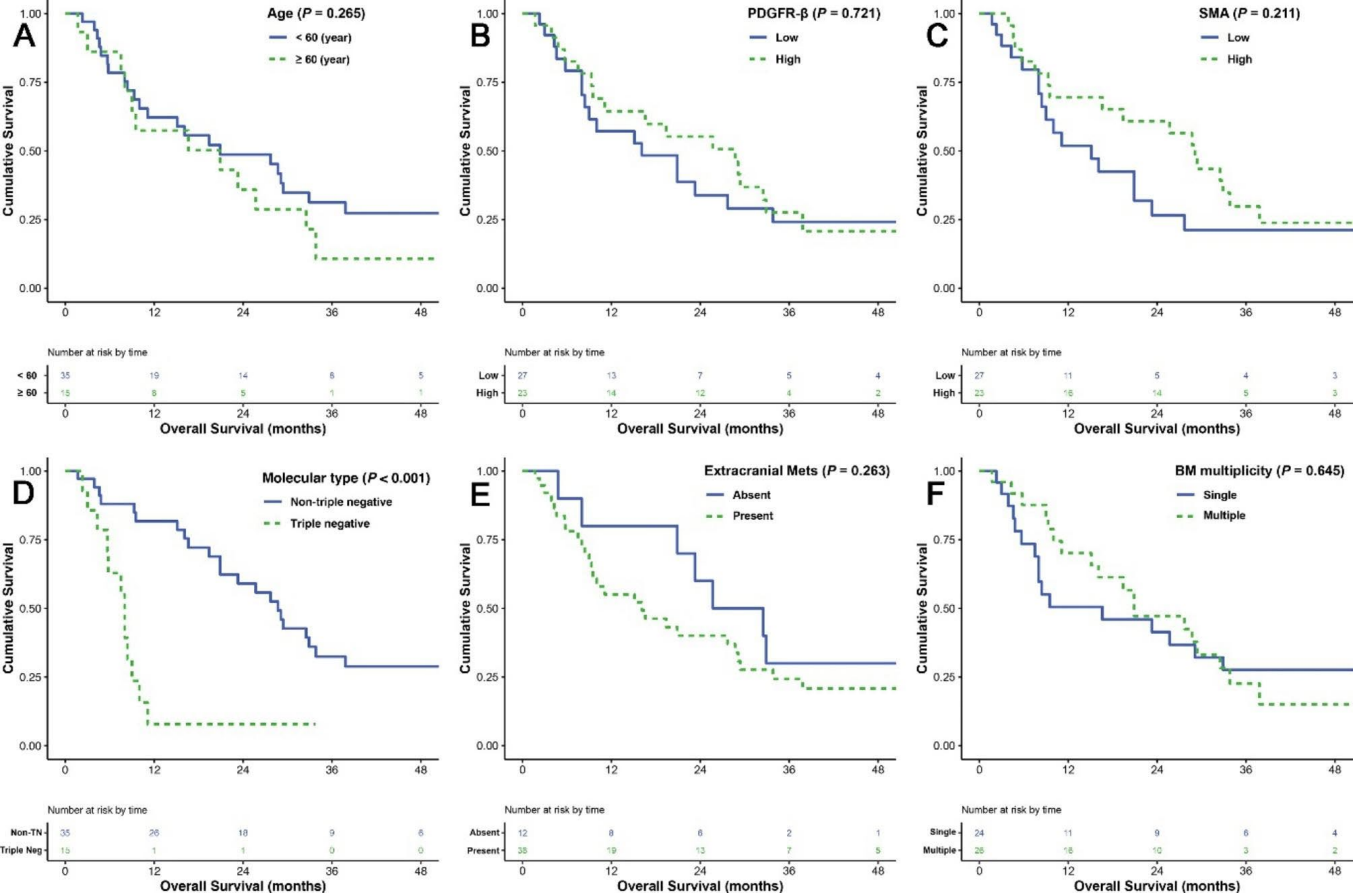
Overall survival (OS) analyses using the Kaplan-Meier estimator and log-rank test were performed according to age (**A**), PDGFR-β expression (**B**), α-SMA expression (**C**), molecular subtype of BCBM (**D**), presence of extracranial metastasis (**E**), and multiplicity of BM lesions (**F**)

In OS analysis (Fig. 4; Table 5), only TN molecular subtype was related to significantly short OS period by univariate analysis (*p* < 0.001) and was also a significant predictor of shorter OS in BCBM patients by multivariate analysis (*p* < 0.001, HR 5.992, 95% CI 2.513–14.285). The expression of PDGFR-β or α-SMA was not significantly correlated with OS. In a view of CAF marker expression, the overall finding shows that low expression of PDGFR-β and α-SMA in stromal CAFs could be considered as a predictor of local recurrence in resected BM.

**Table 5:** Univariate and multivariate analysis for overall survival (OS) predictors in 50 patients with BCBM

Characteristics		No.	Mean (months)	P-value (univariate)	P-value (multivariate)	Hazard ratio	95% CI
Age (year)	< 60	35	31.5	0.265	0.054	1	0.987–4.162
	≥ 60	15	21.6			2.027	
Sex	Female	49	---	---	---		
	Male	1	---				
Molecular type	Non-TN	35	35.0	< 0.001	< 0.001	1	2.513–14.285
	T**N**	15	9.1			5.992	
CAF pattern	Type B	20	29.3	0.498	0.238	1	0.306–1.343
	Others	30	25.9			0.641	
PDGFR-β	Low	27	27.4	0.721	---		
	High	23	27.8				
α-SMA	Low	27	22.3	0.211	---		
	High	23	32.6				
Tumor location	Supratentorial	37	27.6	0.607	---		
	Infratentorial	13	28.2				
Extracranial Mets	Absent	12	38.3	0.263	---		
	Present	38	24.4				
BM symptom	Mild to moderate	31	30.4	0.457	---		
	Severe	19	24.6				
KPS	≥ 80	32	30.6	0.454	---		
	< 80	18	23.6				
BM multiplicity	Single	24	26.9	0.645	---		
	Multiple	26	26.5				
BM development	Metachronous	49	---	---	---		
	Synchronous	1	---				
BM size	< 4 cm	22	31.2	0.643	0.427	1	0.639–2.879
	≥ 4 cm	28	25.5			1.357	
Peritumoral edema	Mild to moderate	27	26.1	0.791	---		
	Severe	23	30.7				
Tumor nature	Solid	27	33.7	0.197	---		
	Cystic or mixed	23	22.1				
Resection degree	Partial to subtotal	12	20.8	0.442	---		
	Gross total	38	29.8				
Postoperative RT/GKS	Absent	16	19.2	0.153	0.111	1	0.265–1.146
	Present	34	31.8			0.551	

BC; breast cancer, BM; brain metastasis, α-SMA; alpha-smooth muscle actin, CAF; cancer-associated fibroblast, GKS; gamma knife radiosurgery, KPS; Karnofsky performance score, Mets; metastasis, PDGFR-β; platelet-derived growth factor receptor-beta, RT; radiation treatment, TN; triple negative

## Discussion

Metastases remain a leading cause of drug resistance and tumor-induced death in BC patients [20]. Therefore, even though advanced chemotherapy based on molecular biology have improved patient survival, the development and trial of new treatment strategy have been continued with an aim of overcoming mechanisms of drug resistance and tumor relapse [21]. The TME plays a fundamental role in tumor development and progression. The study of TME might provide an insight into the novel therapeutic strategies. The fibroblasts are the most abundant cells in TME of solid tumors, such as BC, and are considered the most important elements in TME [22]. Some studies have investigated the role of CAFs in the primary site for tumor progression and distance metastases in BC [23–26]. Among the various CAF markers studied in BC [15], PDGFR-β and α-SMA were found to be important markers dividing the subpopulation of CAF. In particular, it has been reported that PDGFR-β plays an important role in identifying vascular CAF and its origin [27]. It has been reported that α-SMA can be used as an important marker for the subpopulation of CAFs associated with immune evasion of BC [28]. In addition, PDGFR-β was also identified as a marker of CAF, which plays an important role in forming the immunosuppressive TME of BC [29]. However, several studies have been conducted on PDGFR-β and α-SMA as CAF markers in the primary BC, but few studies have been conducted on PDGFR-β and α-SMA in BCBM. The present study aimed to investigate the clinical influence of metastatic site CAFs, especially with high expression of PDGFR-β/α-SMA, on clinical characteristics and prognosis of BCBM patients.

As CAF profiles are modified by reciprocal interaction with cancer cells, each CAFs might express various characteristics in correlation with different features of cancer cells [30]. Our results disclosed differential expression of PDGFR-β and α-SMA in the stroma of the metastatic site according to the molecular subtype of BC. Specifically, the expression of PDGFR-β was relatively low in the TN type. Our study is in line with a previous study which reported that low PDGFR-β expression in the primary site was more likely to be observed in the TN type [31]. Survival rates of BC patients were affected by the molecular subtypes of BCBM, the same as our data [32]. Furthermore, we observed that a higher expression of PDGFR-β or α-SMA indicated a good prognosis, particularly for RFS of patients with BCBM. CAF-related proteins also play an intrinsic role in tumor progression or suppression [33]. Some studies have supported that increased CAF protein subset in the TME tends to prolong the survival of cancer patients [12, 14]. We supposed that one of the heterogeneous CAFs subtypes, especially with high expression of PDGFR-β/α-SMA in BCBM may repress recurrence of tumor in the resection area and was sparsely related with TN, the most aggressive cancer cell subtype. However, regarding OS, the expression of the two markers had no statistical significance, and rather, the survival rate tended to be somewhat longer in the high expression groups. It is presumed that there are multiple factors for the different trends of RFS and OS in relation to the expression of these markers. Other than the fact that the TN molecular subtype acts as a very strong factor in determining the survival rate of BC patients, it is presumed that this may be the result of a complex action of various factors constituting the TME. The definite role of CAF components with regard to the survival rates of BC patients needs to be clarified in the future studies from a larger cohort of patients.

Usually, PDGFR-β and α-SMA are expressed in the stromal fibroblast and help in tissue remodeling, collagen turnover, and the wound healing process [34, 35]. Particularly, the activation and proliferation of fibroblast and production of ECM elements, known as a desmoplastic reaction, represents aspects of cancer progression and clinical outcomes in BC [24]. In the present study, there was a significant correlation between tumor nature (solid or cyst) and the expression of PDGFR-β/α-SMA. The expression of PDGFR-β/α-SMA in BCBM may also be interrelated with desmoplastic reactions. Even more, altered mechanical features of the TME such as ECM stiffness cause fibrosis by developing mechano-activation of fibroblasts. Therefore, it implies that differential expression of PDGFR-β/α-SMA according to tumor natures is correlated with desmoplastic features of BC and different mechanical stress of TME, regardless of the prognosis [36].

Previous studies have classified CAFs subtypes according to morphologic characteristics or molecular profiles [29, 30]. In the present study, the distribution of CAFs varied according to morphologic differences and could be grouped into three patterns (A, B, C). The pattern B type’s CAFs encased individual cancer cells or intermingled closely with small tumor nests. In CAFs in pattern B types, the expression levels of PDGFR-β and α-SMA were relatively high. The morphologic pattern is often related to the molecular profile and further to the functional aspect. However, unlike the correlation between the expression of PDGFR-β/α-SMA and tumor progression, there was no significant connection between our morphologic subtypes and patient clinical features. Therefore, a more detailed and suitable morphologic classification of CAFs is required for a more cost-effective prediction of prognosis with simple tissue analysis.

It is hard to anticipate the prognosis of patients with BCBM, although several prognostic factors have been suggested, which are associated with poor prognosis. As age, KPS, tumor subtypes, extracranial metastasis, and the number of BM are associated with survival in patients with BCBM, these factors were used to identify patients with good or bad prognosis [37, 38]. However, the above mentioned factors did not show a significant association with poor prognosis in the present study, except for tumor subtypes. Those factors only showed a tendency to relate to RFS, and PDGFR-β/α-SMA also showed a tendency to a similar degree. There is a low possibility for PDGFR-β/α-SMA to be a strong prognostic biomarker, further large-scale studies should verify PDGFR-β/α-SMA as feasible biomarkers to help ameliorate predicting the patient’s prognosis with BCBM. Because this was a retrospective study with a relatively small number of patients, there may be a possibility of selection bias. In addition, this study was a simple clinico-radiological and pathological investigation using only two CAF markers. To determine the exact role of CAFs for patients with BM from BC, a further prospective study with a large sample size and more detailed information on various CAF markers is needed.

## Conclusions

Our study showed different expressions levels of PDGFR-β and α-SMA in stromal CAFs of BMs according to molecular subtypes of BC. The expression of the markers was lower in TN patients, the most aggressive molecular subtype, compared to other Luminal or HER2^+^ BC patients. Besides, this study investigated the correlation between stromal CAFs with high PDGFR-β/α-SMA expression and favorable prognosis of BCBM patients. More aggressive BC could be capable of metastasis to the brain, and recurrence after surgical resection, with CAFs subtype with low PDGFR-β/α-SMA expression. The present study aimed to show the clinical influence of metastatic site CAFs, in connection with biomarkers, PDGFR-β and α-SMA. Owing to the limitations of this study, further research on the exact role of CAFs is needed to provide an insight into the development of new therapeutic strategies for BCBM patients with a dismal prognosis.

## Electronic supplementary material

Below is the link to the electronic supplementary material.

Supplementary Material 1

## Data Availability

The datasets used and analyzed during the current study are available from the corresponding author on reasonable request.

## Abbreviations

α-SMA: Alpha-smooth muscle actin
BC: Breast cancer
BM: Brain metastasis
CAF: Cancer-associated fibroblast
IHC: Immunohistochemistry
PDGFR-β: Platelet-derived growth factor receptor-β
TME: Tumor microenvironment
