# Supplementary material for: Analysis of stromal PDGFR-β and α-SMA expression and their clinical relevance in brain metastases of breast cancer patients
Source: BMC Cancer. 2023 May 22;23:468. doi: 10.1186/s12885-023-10957-5 (PMC10201734; doi:10.1186/s12885-023-10957-5)
Supplement: Supplementary file 1 — Supplementary Material 1 [file 12885_2023_10957_MOESM1_ESM.docx]

Supplementary Table 1. Comparison of immunohistochemical expressions of PDGFR-β in BM and matched primary BC samples.

|  | | Matched primary BC | | | Total | *Kappa* value |
| --- | --- | --- | --- | --- | --- | --- |
|  |  | Score 1 | Score 2 | Score 3 |  |  |
| BM | Score 1 | 0 | 3 | 2 | 5 | 0.170 |
|  | Score 2 | 0 | 4 | 2 | 6 |  |
|  | Score 3 | 1 | 1 | 5 | 7 |  |
|  | Total | 1 | 8 | 9 | 18 |  |

BC; breast cancer, BM; brain metastasis, CAF; cancer-associated fibroblast, PDGFR-β; platelet-derived growth factor receptor-beta

Supplementary Table 2. Comparison of immunohistochemical expressions of α-SMA in BM and matched primary BC samples.

|  | | Matched primary BC | | | Total | *Kappa* value |
| --- | --- | --- | --- | --- | --- | --- |
|  |  | Score 1 | Score 2 | Score 3 |  |  |
| BM | Score 1 | 0 | 0 | 4 | 4 | 0.148 |
|  | Score 2 | 0 | 4 | 1 | 5 |  |
|  | Score 3 | 0 | 3 | 6 | 6 |  |
|  | Total | 0 | 7 | 11 | 18 |  |

BC; breast cancer, BM; brain metastasis, α-SMA; alpha-smooth muscle actin, CAF; cancer-associated fibroblast

Supplementary Table 3. Comparison of immunohistochemical expressions of PDGFR-β and α-SMA in stromal CAFs of 18 primary BC samples.

|  | | α-SMA | | | Total | *Kappa* value |
| --- | --- | --- | --- | --- | --- | --- |
|  |  | Score 1 | Score 2 | Score 3 |  |  |
| PDGFR-β | Score 1 | 0 | 0 | 1 | 1 | 0.091 |
|  | Score 2 | 0 | 5 | 3 | 8 |  |
|  | Score 3 | 0 | 2 | 7 | 9 |  |
| Total | | 0 | 7 | 11 | 18 |  |

BC; breast cancer, α-SMA, alpha-smooth muscle actin; CAF, cancer-associated fibroblast; PDGFR-β, platelet-derived growth factor receptor-beta
